# Supplementary material for: The CXCL10/CXCR3 axis is essential for sustaining immunological dormancy in triple-negative breast cancer
Source: NPJ Breast Cancer. 2026 Jan 30;12:36. doi: 10.1038/s41523-026-00903-6 (PMC12960658; doi:10.1038/s41523-026-00903-6)
Supplement: Supplementary file 1 — Supplementary Information [file 41523_2026_903_MOESM1_ESM.pdf]

Supplementary Information

**Supplementary Data 1**	**Transcriptomic profiling of D2.0R versus D2A1 cells in culture**
**Supplementary Data 2**	**Transcriptomic profiling of D2.0R-*shCxcl10* versus D2.0R-*shCTL* cells**
**Supplementary Data 3**	**Gene list for D2.0R dormancy signature, MR20 dormancy signature, the CXCL10-induced dormancy signature, and its human orthologs**

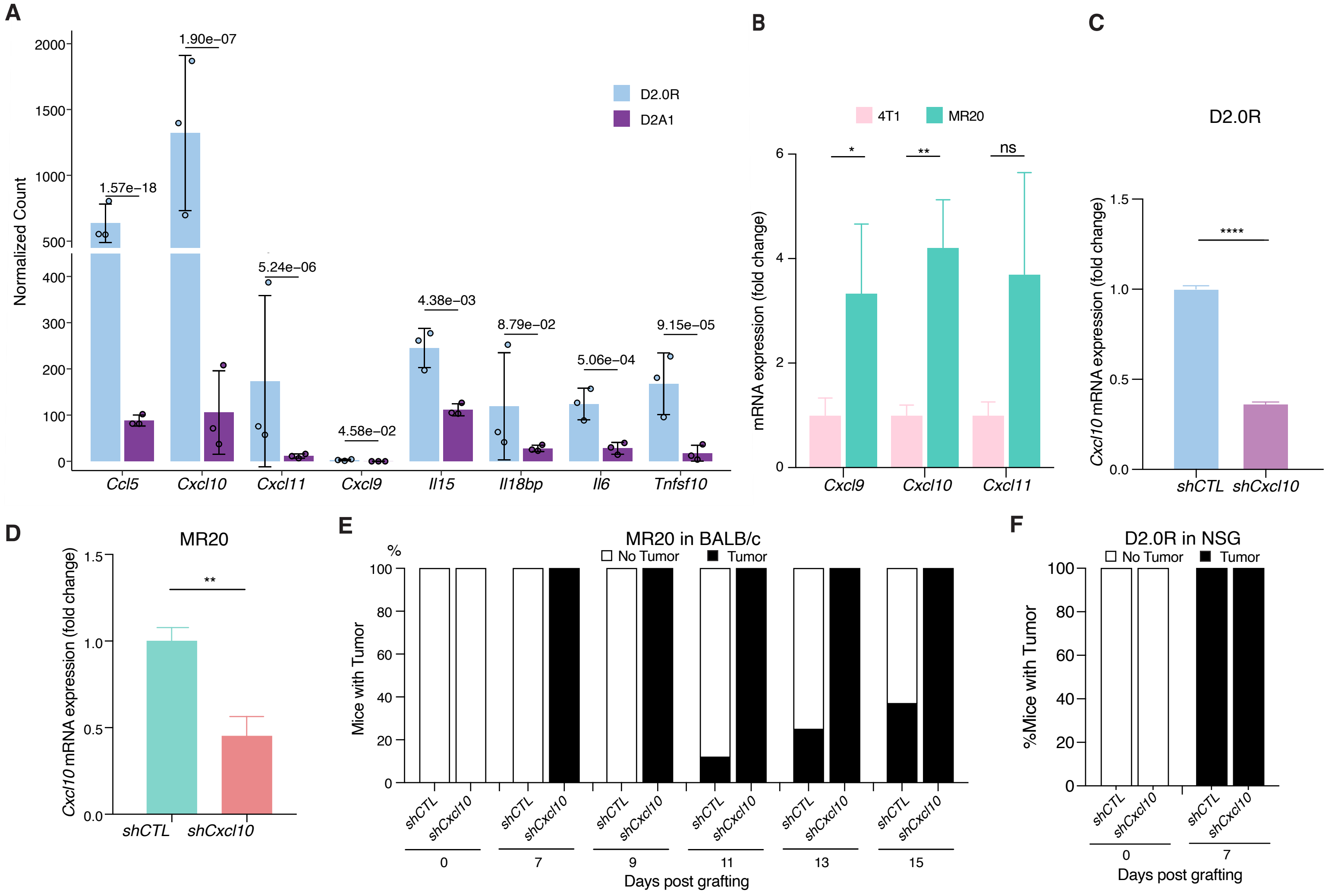

Supplementary Figure 1. Selected gene expression analysis in D2A1 vs D2.0R and effect of *Cxcl10* silencing D2.0R cells on tumor incidence

A Normalized RNA-seq counts of the indicated ligand coding genes that belong to the INTERFERON ALPHA and GAMMA RESPONSE pathways and are differentially expressed between D2.0R and D2A1 cells.

B Relative mRNA expression of *Cxcl9*, *Cxcl10* and *Cxcl11* in MR20 and 4T1 cell lines examined by RT-qPCR. n= 2-3/group.

C, D Validation of lentiviral-mediated *Cxcl10* silencing in D2.0R cells **(C)** and MR20 **(D)** measured by RT-qPCR. The tumor cells infected with lentivirus carrying non-targeting control vector were used as control (*shCTL*) and expression level set to 1. n = 3-4/group.

E Percentage of immune-competent BALB/c mice developing tumors over time following orthotopic implantation of *shCTL*- or *shCxcl10*-transduced MR20 tumor cells. n= 7-8/group.

F Percentage of immune-deficient NSG mice developing tumors over time following orthotopic implantation of *shCTL*- or *shCxcl10*-transduced D2.0R tumor cells. n= 7-8/group.

For panel **A**, the data are presented as mean ± SD, and *P* value are calculated using Wald test. The data in **B-D** are represented as mean ± SEM, and P values were calculated unpaired two-tailed Student's t test. ns, not significant; *, $P < 0.05$; **, $P < 0.01$; ****, $P < 0.0001$.

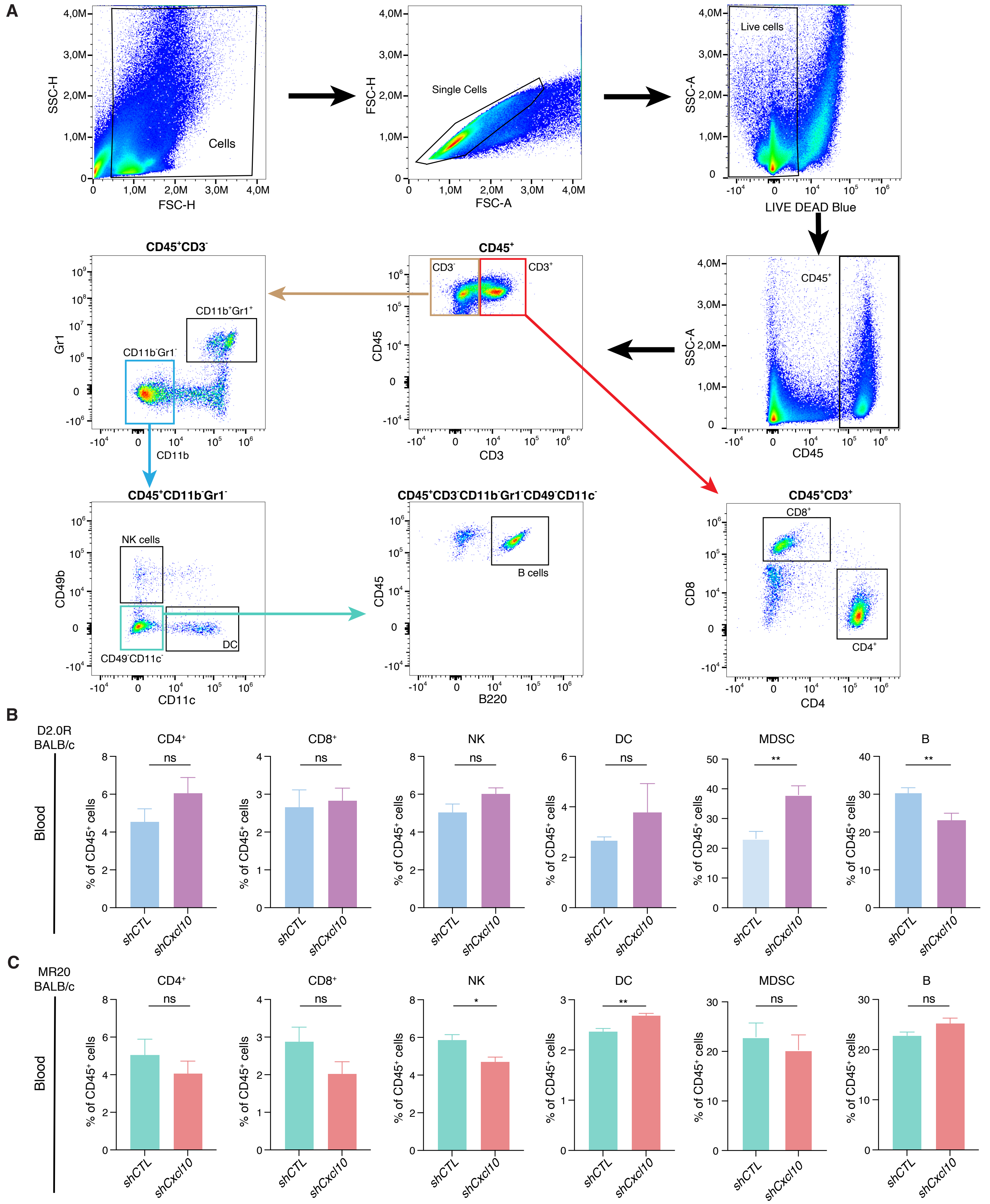

Supplementary Figure 2. Effect of *Cxcl10* silencing D2.0R and MR20 cells on immune cell populations in peripheral blood

A Gating strategy for the identification of immune cell populations by flow cytometry

B, C Fraction of immune cell populations in peripheral blood of BALB/c mice orthotopically injected with *shCTL* or *shCxcl10* tumor cells derived from D2.0R **(B)** or MR20 **(C)**, as determined by flow cytometry analysis. Results are expressed as percentage of the indicated cell populations within CD45$^+$ cells. n= 6-8/group. The data are represented as mean ± SEM, and *P* values were calculated unpaired two-tailed Student's t test. ns, not significant; *, *P* < 0.05; **, *P* < 0.01.

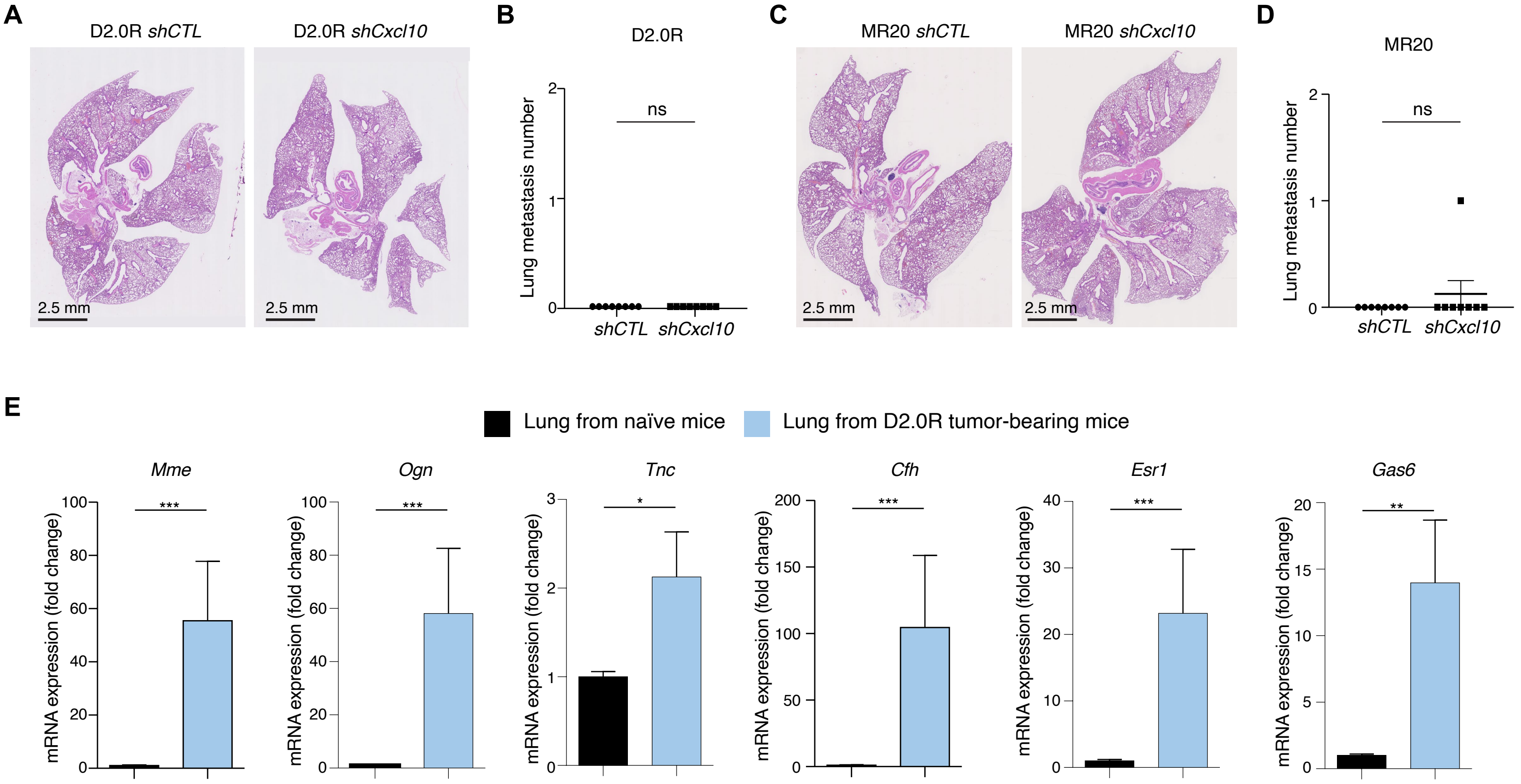

Supplementary Figure 3. *Cxcl10* silencing does not impact lung metastasis

A-D Representative histological images of lung (H&E staining) and corresponding quantifications of metastatic burden from BALB/c mice orthotopically injected with D2.0R-*shCTL* or D2.0R-*shCxcl10* tumor cells (**A** & **B**) and MR20-*shCTL* or MR20-*shCxcl10* tumor cells (**C** & **D**). Scale bar: 2.5 mm. n = 8/group.

E Relative mRNA expression of D2.0R tumor-related genes, *Mme, Ogn, Tnc, Cfh, Esr1* and *Gas6*, in the lungs of naïve mice and lungs of D2.0R cells-injected into 4th mammary fat pad of BALB/c mice. Expression levels in naïve mice are adjusted to 1. n= 7-9/group.

Data are represented as mean ± SEM. *P* values were calculated using Mann-Whitney test (**B** & **D**) or unpaired two-tailed Student's t test (**E**). ns, not significant; *, *P* < 0.05; **, *P* < 0.01; ***, *P* < 0.0005.

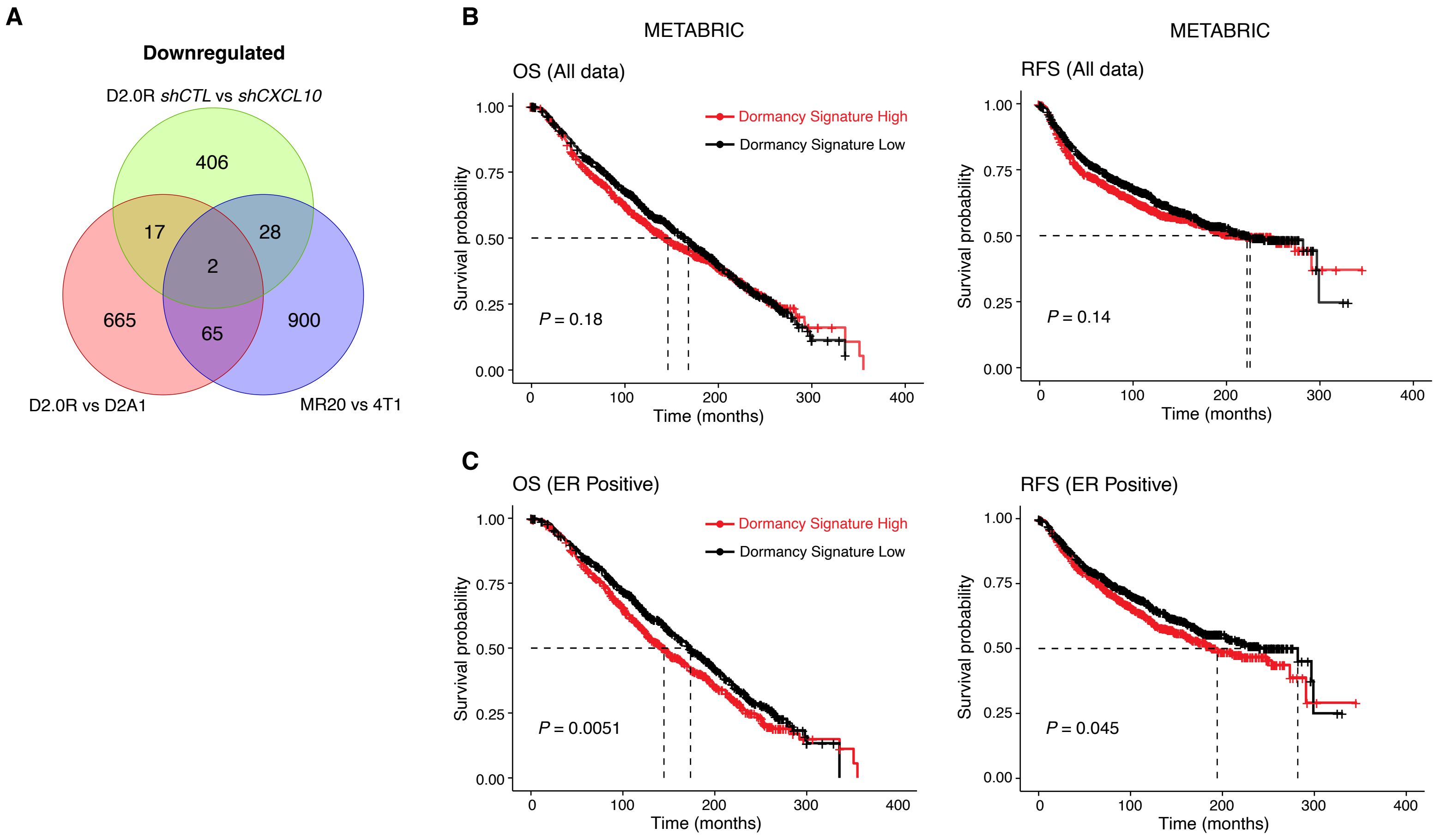

Supplementary Figure 4. CXCL10-induced dormancy signature expression in breast cancer patients and effect on OS and RFS

A Venn diagram showing common genes that are downregulated in dormant D2.0R and MR20 cells compared with their respective non-dormant controls and upregulated in D2.0R upon *Cxcl10* knocking down (downregulated when comparing D2.0R *shCTL* vs *shCxcl10*).

B, C Kaplan-Meier curves showing OS and RFS for all patients **(B)** and ER^+ subsets patients **(C)** according to high or low expression of human orthologue of Dormancy Signature in METABRIC data sets. *P* values were calculated using log-rank test.
